# Barriers to Help-Seeking for Sexual Violence Among Married or Cohabiting Women in Ghana

**DOI:** 10.1177/10778012211060861

**Published:** 2021-12-13

**Authors:** Gervin A. Apatinga, Eric Y. Tenkorang

**Affiliations:** 1 Department of Geography and Planning, University of Saskatchewan, Saskatoon, Canada; 2 Department of Sociology, 98997 Memorial University of Newfoundland, St. John's, Canada

**Keywords:** Africa, barriers model, Ghana, married or cohabiting women, sexual violence

## Abstract

While sexual violence against women has gained attention in sub-Saharan Africa, research examining help-seeking remains limited. Scholarship on barriers to help-seeking among sexually abused married or cohabiting women is particularly lacking. We used the barriers model and held 15 in-depth interviews with sexually abused Ghanaian married or cohabiting women to examine help-seeking behaviors. Participants identified multiple barriers to help-seeking, including financial difficulties, lack of social support, and stigma. The results corroborate the barriers model's formulation of the challenges faced by female survivors in reporting violence. They indicate the need to improve laws to promote help-seeking among women with experiences of sexual violence.

## Introduction

Sexual violence is “any sexual act, attempt to obtain a sexual act, or other act directed against a person's sexuality using coercion, by any person regardless of their relationship to the victim, in any setting including but not limited to home and work” (Jewkes et al., 2002, p. 149). As such, sexual violence violates human rights and exposes victims to multiple intersecting vulnerabilities, including but not limited to physical injuries and psychosocial health problems. While men can be victims and survivors of sexual violence, the majority are women, especially female pubescents (Josse, 2010; World Health Organization [WHO], 2014). In sub-Saharan Africa, for example, one in three women experiences sexual violence at the hands of a male partner during her lifetime (WHO, 2016), while globally, one-third of all women experience severe forms of sexual violence, such as rape, and more than one-third of female pubescents report forced sexual initiation (Gender Studies & Human Rights Documentation Centre [GSHRDC], 2015; WHO, 2013). Most countries have introduced laws to protect against sexual violence (Watts & Zimmerman, 2002; WHO, London School of Hygiene and Tropical Medicine, 2010), including Ghana, with its Domestic Violence Act (732) (Institute of Development Studies, Ghana Statistical Services and Associates [IDS, GSS & Associates], 2016).

Despite the passage of laws, sexual violence against women remains a problem in almost all countries worldwide (Richter et al., 2004; WHO, 2005). Some scholars suggest the problem may be attributed, in part, to a lack of help-seeking among female survivors (Benoit et al., 2015; Brennan & Taylor-Butts, 2008; Luce et al., 2010). Help-seeking occurs when survivors of violence report/disclose their experiences and access a range of services aimed at dealing with the consequences of the abuse. Help-seekers may turn to formal or informal support avenues. Support from family, friends, co-workers, and other members within the network of the survivor is often considered informal, while services offered by bureaucratic organizations, including the police and domestic violence organizations, such as the Ghana Domestic Violence and Victim Support Unit (DOVVSU), are considered formal. Help-seeking, in this article, is defined as accessing the services of formal organizations, including the police and DOVVSU.

The literature suggests many female survivors of sexual violence do not report perpetrators (Brennan & Taylor-Butts, 2008; García-Moreno et al., 2005; Usta et al., 2007). In Ghana, for instance, accumulating evidence indicates the majority of female survivors of sexual and intimate partner violence do not seek help from formal support networks, including law enforcement agencies (Anyemedu et al., 2020; Sedziafa et al., 2016; Tenkorang et al., 2018). These studies reveal high rates of non-help seeking behavior among Ghanaian women, and suggest this silence has implications for the health and well-being of survivors.

While research examining the drivers of non-help-seeking behaviors among female survivors of violence is growing in sub-Saharan Africa (Anyemedu et al., 2020; Liang et al., 2005; Tenkorang et al., 2018), some critical aspects of this important topic remain unexplored. Research focusing on barriers to help-seeking among women who suffer sexual violence in marriage is particularly sparse. This research lacuna is problematic because the literature to date finds these women encounter multiple challenges to reporting the violence to law enforcement agencies (Adu-Gyamfi, 2014; Apatinga & Tenkorang, 2020; Chireshe, 2015). For instance, some evidence suggests the bride price^1^ tradition in many African societies gives men the privilege to control their spouses, including their sexuality and sexual behaviors (Adinkrah, 2011; Amoakohene, 2004; Chirwa et al., 2018). Other authors underscore that in some cultural and ethnic groups, sexual matters are private, and forced sex is not recognized as abusive but as a husband's conjugal right (Amoah et al., 2021; Kidman, 2016). This suggests women who suffer sexual abuse in marriage are unlikely to report to law enforcement agencies such as the police. Yet, help-seeking through these agencies is associated with positive outcomes and considered helpful for female survivors (Anyemedu et al., 2020; Ofstehage et al., 2011; Tenkorang et al., 2016).

In this context, promoting help-seeking behaviors would involve removing or minimizing barriers to reporting sexual violence by examining and understanding them. Contributing to the sparse but nascent literature on sexual violence within intimate relationships, this study examined barriers to help-seeking among married or cohabiting women experiencing sexual violence in the Eastern Region of Ghana.

## Theoretical Framework: Barriers Model

**Figure 1.**
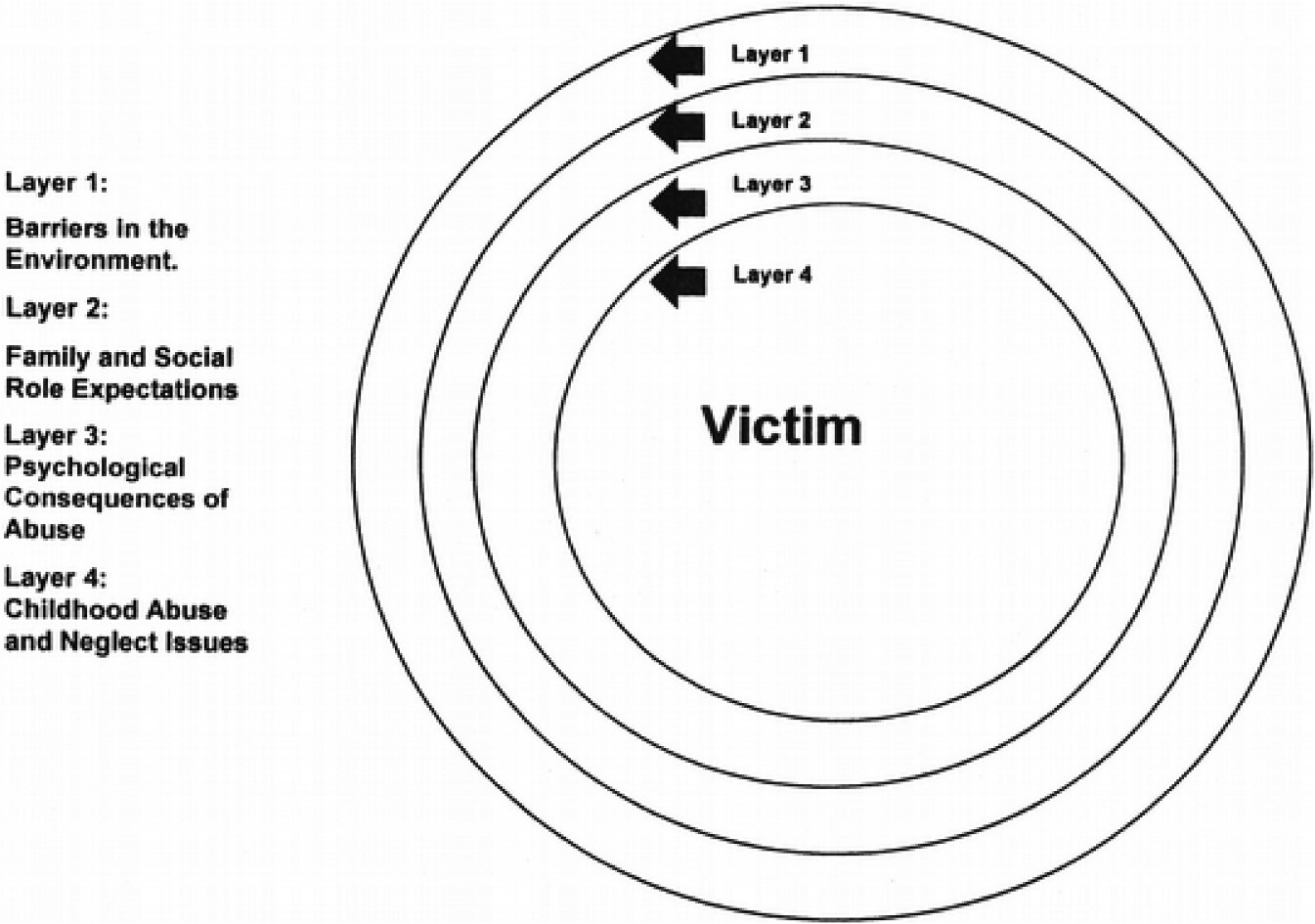
Barriers model.

Source: Grigsby and Hartman (1997).

The theoretical framework of the study is the barriers model. The model was developed to understand barriers to the well-being of survivors of domestic violence, including non-help-seeking behaviors. It emphasizes the removal of these barriers to ensure the safety and security of survivors of violence (Grigsby & Hartman, 1997). The model provides a broad critical lens through which to view the experiences of sexual and intimate partner violence. It offers an alternative conceptual framework for understanding why female survivors of sexual and domestic violence will not seek help to find safety and avoid revictimization. The model places abused women at the center of four aggregate concentric circles (see Figure 1)*,* with each ring representing a layer of barriers to help-seeking.

The outermost circle represents barriers in the environment that trap women in abusive relationships. Barriers in this layer include lack of access to information, unemployment, poverty, financial problems, lack of police assistance, inaccessible mental health services, lack of transportation, and inaccessible legal counsel. For instance, because of the processes involved in seeking legal redress, female survivors who are unemployed and not economically independent may find it difficult to report violence to law enforcement agencies. Similarly, women who are economically dependent on their male partners may have little or no autonomy to report assault. Female survivors who lack information about agencies responsible for dealing with crime are also unlikely to report violence. This suggests the need to provide women with opportunities to improve their economic and social status.

The second ring represents barriers related to family/socialization/social role expectations. Examples include values/beliefs about relationships, identity, values related to abuse, religious beliefs, and family origins of values/beliefs (Grigsby & Hartman, 1997). For instance, in sub-Saharan Africa, women are often expected to help build or protect their families, as they are assumed to be home keepers or caregivers. Internalizing these roles can cause women to shoulder the blame for conflict in a marital relationship. Women who accept blame are unlikely to leave the marriage, as they are expected or would like to stay and fix or rebuild it.

The third layer represents psychological barriers, including defense mechanisms, physical/somatic results, isolation, brainwashing, compliance strategies, and post-traumatic stress disorder (Grigsby & Hartman, 1997). Fear, hyper-vigilance, and a lack of trust are the most frequent end results of abuse (Adu-Gyamfi, 2014; Apatinga, 2019).

Childhood abuse and neglect issues constitute the last layer, and this barrier may trigger other barriers. It includes early messages about abuse, safety, and psychological consequences. Children learn what is expected of them and how they should relate with people and deal with emotions. Family members are considered the most trustworthy people (Anderson, 2014). If children are victimized by someone in the family, they tend to assume anyone can abuse them. Consistently abused children learn there is no escape. They accept that danger is always present. Thus, women who have been abused during childhood are more likely to tolerate violence, making escape from an abusive relationship difficult.

It is important to note that while some victims may encounter only one of these barriers, there is a high possibility of experiencing all barriers or a combination of two or three (Grigsby & Hartman, 1997). We employed this model to unpack the help-seeking behaviors of women with experiences of sexual violence in the Lower Manya Krobo District in the Eastern region of Ghana.

## Methods

### Geographical Context

Data were obtained from the Lower Manya Krobo District (LMKD), one of the 26 administrative areas in the Eastern Region of Ghana. The locality was carved out of the former Manya Krobo District, and Odumase Krobo is the capital city. The area covers a total landmass of 591 sq.km, constituting approximately 3.28% of the entire land area of the Eastern Region. The municipality is peri-urban and has a population of approximately 89,246 (IDS, GSS & Associates, 2016). There are more females (53.5%) than males (46.5%) (Ghana Statistical Service, 2014). Although there are other tribes, residents are mainly Krobos (Ghana Statistical Service, 2014). The majority are Christians, with a few Muslims and traditional worshippers (Ghana Statistical Service, 2014). The people are predominantly farmers and traders. The area is endowed with natural resources such as limestone and has several tourist sites, including beautiful landscapes, the Kpong airfields, and beach resorts (IDS, GSS & Associates, 2016). Yet, poverty is endemic, especially among women (Otoo et al., 2009). Even employees in the government sector earn less than the minimum wage of US$2. The municipality is reported to have the highest rate of unemployment in the Eastern Region of Ghana (Ghana Statistical Service, 2013).

While women are fundamental to the general socio-economic development of the LMKD, they face challenges in both private and public sectors, limiting their contribution to the growth of the local economy. Gender-based violence is one of the key challenges to exploiting the full development potential of women. As elsewhere in Ghana, in the LMKD, violence against women is widespread and occurs in several forms, including verbal, physical, and sexual violence (IDI, GSS & Associates, 2016; Tenkorang et al., 2013). Although women experience violence from several types of perpetrators, such as family relations, community members, and strangers, the most common perpetrators of violence are current or former intimate partners (Sedziafa et al., 2017; Tenkorang et al., 2013). Evidence has shown the Eastern Region has one of the highest rates of intimate partner violence in Ghana (IDI, GSS & Associates, 2016). Sex without permission is reported by 0.7% of the population in the Eastern Region; only the Central Region in Ghana has a higher percentage (Ghana Family Life and Health Survey [GFLHS] 2015 cited in IDI, GSS & Associates, 2016). Other forms of male-perpetrated sexual violence reported in this region include inappropriate sexual comments (0.8%), sexual touching (0.8%), sex without protection (0.6%), and sex because of fear (0.6%) (Ghana Family Life and Health Survey [GFLHS] 2015 cited in IDI, GSS & Associates, 2016). Several researchers have identified women in the LMKD as highly susceptible to intimate partner violence, including sexual violence (Sedziafa et al., 2016; Sedziafa et al., 2017). It is often argued that the reported and documented data represent a small number of the actual cases of sexual and intimate partner violence, a problem attributed to the non-disclosure or underreporting of violent incidents.

Despite the introduction of policies and programs by Ghanaian policymakers to help protect against abuse in domestic interactions, the majority of female victims, including those in the LMKD, rarely seek help from formal support units, allowing many perpetrators to go unpunished (Boakye, 2009; Boateng, 2015). For instance, formal resources such as the police and DOVVSU are available in the LMKD to address cases of violence, yet the majority of female victims seldom access these services. In particular, women in the context of marriage or cohabitation rarely disclose their experiences to formal support units (Sedziafa et al., 2017).

Given this background, the Lower Manya Krobo Municipality in the Eastern Region of Ghana provides an interesting case for analyzing the barriers to reporting sexual violence among married or cohabiting women.

### Participant Recruitment

Data for this study were obtained from a larger research project that recruited women in the LMKD who had experienced intimate partner violence, including sexual abuse, in their marriages. Before data collection, the chief and his elders (gatekeepers of the community) were informed about the aim of the study and were supportive of the research. We then identified women survivors of sexual intimate partner violence with the help of local opinion leaders. After we obtained their consent, these women were recruited as participants. Through the snowballing method, other eligible participants were recommended. Eligibility criteria were women who had experienced sexual intimate partner violence, were at least 18 years old, and were legitimately married or had been cohabitating for a minimum of one year. Ultimately, 15 women shared their experiences and perspectives on violence, including barriers to reporting sexual violence.

### Data Collection

The main tool for data collection was face-to-face in-depth interviews, as interviews help to gain a deeper understanding of the main issues (Boyce & Neale, 2006). Women who had consented were briefed about the purpose of the study, and a suitable time and location were suggested by participants for the interviews. Trained female research assistants moderated the individual interviews in a sociable manner to help participants feel relaxed and to prevent them from being frightened. These research assistants were trained by the second author at the University of Ghana before data collection. The interviews were held at safe locations suggested by the participants. Participants were asked the same set of questions, and further probing was intermittently carried out by the interviewers to clarify ideas and responses pertaining to male partner violence and non-help-seeking behaviors. A flexible interview guide was used to direct the interviews, covering the following themes: personal details, the contexts of violence, the reasons for violence, the consequences of violence, and help-seeking behaviors. Before data collection, the interview questions were pre-tested with a sample of five respondents, and the questions were modified based on the pre-test results. Although the interview guide was prepared in English, it was interpreted in Krobo (widely spoken local dialect) by the research assistants for participants who were unable to speak and understand English. Translation was crucial, as it enabled participants to easily understand and respond meaningfully to questions. As sharing a personal experience of violence can be disturbing, counselling services were made available for participants who felt intimidated by recounting their ordeals. The interviews lasted between 30 min and 1 h, on average, to ensure a meaningful and productive conversation. With the consent of participants, all the interviews were audio-recorded to capture every detail and to avoid the distortion of responses. Pseudonyms were used to protect and anonymize participants’ personal information.

### Ethical Considerations

The data for this study were obtained from a larger research project approved by the Inter-disciplinary Committee on Ethics in Human Research of Memorial University, Canada, and the University of Ghana Ethics Committee for Humanities. After gaining ethics approval, the principles of anonymity, confidentiality, and the right to end participation in the study without consequences were assured and strictly observed. The rights of participants were respected, an informed consent sheet detailing the purpose of the study was made available to each participant, and consent was sought before all interviews. For instance, an interviewer asked a participant, “Please, am I permitted to talk to you?” In turn, the participant would answer, “Yes, you have my permission.” All the interview transcripts and other data were alphanumerically coded when the scripts were being analyzed. Participants were allowed to choose their own place and time for the interviews so that no third party would know they had participated in the study. In short, the research team adhered to the safety and ethical recommendations for research on intimate partner violence by the World Health Organization (WHO, 2001).

### Data Analysis

We used thematic analysis to analyze the qualitative data because of its emphasis on working with data, systematic and objective coding, organizing data, and searching for patterns to develop themes (Braun & Clarke, 2006). Before analysis, interviews conducted in the local language (Krobo) were translated back into English by the research assistants during the data transcription. The interviews were back-translated to identify the correct version of the text and check for misrepresentation (Temple & Young, 2004). The transcribed interviews were analyzed through manual coding. We first read the transcripts many times to become acquainted with the pattern of responses and to understand the trend of narratives pertaining to the underreporting of sexual and intimate partner violence, looking for emerging topics based on the aim of the study. We used colored pens and tables to highlight and summarize information on the nondisclosure of sexual violence, highlighting related issues and responses in the same color. The data were reexamined multiple times to clarify issues and avoid the misrepresentation of responses. The first author, supervised by the second author, assigned codes to corresponding ideas and responses. We were reflexive in this process by ensuring that our gender identities as males socially and culturally trained in a patriarchal environment did not interfere with women's experiences and perspectives of sexual violence. The codes identified important categories and sub-categories, and these were synthesized into themes. We reviewed the themes a number of times and used those more closely related to the research objective as final themes to present the findings. In what follows, some of the participants’ comments are used to support the findings.

## Findings

### Participants

The sample included 15 women aged 25 to 65 years, with an average age of 42.86 years. Almost all the women were married or cohabiting at the time of data collection. Apart from one woman who identified as Ewe, all were Krobos. Their educational status differed: two women finished primary education, one completed tertiary education, four attained some level of secondary education, and a significant number did not complete or attempt any level of education. All the women were Christians, and the number of children born to each woman ranged from one to ten. The majority were petty traders; a few were subsistence farmers, and one woman identified as a professional teacher.

### Barriers to Help-seeking Among Sexually Abused Married or Cohabiting Women

Participants identified many barriers to help-seeking. These are discussed under the following broad themes based on the barriers model: barriers in the environment, barriers due to family/socialization/role expectations, and psychological barriers and childhood abuse and neglect issues.

#### Barriers in the Environment

##### High Financial Cost of Help-seeking

Participants said a lack of financial resources made it difficult to report sexual assaults. Many mentioned the high cost of seeking help as a reason for not involving the police. If they were employed in low-paying economic activities, they could not afford to seek legal redress, nor could they pay for transportation and other services. One woman recounted her experience:

I know many women are raped by their husbands. It has happened to me before, but due to financial challenges, it was difficult to report it at the appropriate place. My father was dead, and my mother was sick; therefore, there was no support from my family. I went to the Dumase-Krobo^2^ police station in the evening after the incident occurred, but I was directed to Akuse police station, where domestic violence issues were being addressed. But even transportation to the place was a problem, and that discouraged me from pursuing the case. (Etonam, 25 years, petty trader, primary education, three children, Ewe)

Another participant stated:

I am financially handicapped, and my family is not rich, so it was difficult reporting him [husband] to the police. He even said it himself that I cannot report him because I do not have money. (Memunatu, 56 years, petty trader, form 4-level education, two children, Krobo)

##### Child Support and Well-being

Some women declined to report their abusive husbands because they feared for the safety and well-being of their children. Most were worried that they would be unable to support their children alone, as they were economically dependent on their husbands. They believed the non-disclosure of sexual assaults and abuse to legal authorities was worthwhile for the sake of their children's security. This type of concern is demonstrated in the following accounts:

I have kids with him, and when you go to report and he is arrested, all the burden of taking care of the children will be on you. I cannot do it alone. It is not easy to take care of children when you are a single parent. Taking care of children involves a lot. It needs a lot of money, and I am only a petty trader, which hardly gives me enough money. (Fati, 47 years, farmer, no education, eight children, Krobo)

I do not want to be a single parent and do not want my children to be fatherless, since this will have dangerous effects on them, especially the boys. It is difficult for a woman to bring up a boy child, so the man is needed to bring up the children, especially the male children. (Wepia-28 years, petty trader, secondary education, three children, Krobo)

In the following narrative, Latifa, who suffered serious injuries following sexual violence, explained how she refused to involve or contact the police because of her love for her children:

I had wanted to report him and leave but decided to stay because of my children. Some of the beatings affected me so much that I had to go buy drugs to treat myself. Sometimes, I feel very sick the morning after the night he beats me because of sex. There was an instance where he got very angry and took the iron that was lying on the ironing bed close to the bed and threw the iron at me. It hit my head, and I felt a sharp pain in my head. It became so severe that I had to go to the hospital. I went and was treated, but occasionally, I feel the pain. It has caused many effects on my health, but I have no option other than to stay since I do not want another woman to come and maltreat my children when I am not around. (Latifa, 26 years, petty trader, primary education, three children, Krobo)

Some even said they preferred to die at the hands of their husbands rather than report sexual assaults and abuse to law enforcement agencies, mainly to protect the welfare of their children:

As for my husband, even if he ever cuts me with a knife, I will nurse the wound until it is healed. I could not even look at my children and still report their father to the police. This can even lead to divorce, and all my children could become wayward. No matter how much my husband misbehaves towards me, I never report to the police. The elders of both families would come in to settle the issue, and that would be it. (Ayishetu, 38 years, mother of four, small-scale trader)

##### Concerns About the Justice System

A number of participants expressed concerns about the justice system, especially the police force. They explained sexual violence is not given the attention it needs in their community. Some thought reporting sexual abuse might cause more harm than good, as the perpetrator would likely escape punishment, leaving the survivor without justice. They voiced disappointment in the police force and complained corruption was rampant, preventing action against domestic violence. They strongly believed the lack of punishment for abusive husbands created grounds for repeated assaults:

If you report your husband, he will give them [police] money and they will let him go. So, you can't trust the police. (Fati, 47 years, farmer, no education, eight children, Krobo)

When you report a case to the police, they will be postponing it. You will be paying for transportation to the police station all the time, and all this is a waste of time and resources. (Asana, 55 years, petty trader, form 4-level education, three children, Krobo)

#### Barriers Due to Family/Socialization/Role Expectations

##### Lack of Social Support

Another driving force behind women's non-help-seeking behavior was a lack of social support in the community. Sexually abused women were often discouraged by family and friends from reporting their husbands to law enforcement agencies. Many survivors said they experienced pressure from their immediate in-laws and external relatives to desist from pressing charges. Reporting their husbands had significant implications. The following comments explain how the lack of social support impeded women's decisions to report abusive husbands:

I once tried to report my husband to the police. I went to the Odumase police station to report my husband's actions towards me. I was asked by the police officers to go to Akuse,^3^ where domestic violence issues were addressed, but my family later discouraged me. (Latifa, 26 years, petty trader, primary education, three children, Krobo)

I planned to have him [husband] arrested, but people came in to plead, saying if I have him arrested, then that is like literally ending the marriage. I was going to report, and friends came to beg me not to report, so they were going to give me money to go to the hospital. But I told them the money is not necessary, and I am not going to report anymore. (Esi, 28 years, storekeeper, tertiary education, one child, Krobo)

Some participants explained that in Krobo areas, when a woman reports an assault, she is criticized for complaining:

In Ghana, the elderly do not like to talk about issues in marriages. Even when you go home to report such issues to parents, they say you should be patient and that things will get better. When you talk about divorce, nobody seems to like it, so most people believe that even if the man is misbehaving, you should still stay with him, and things will change one day. Sometimes they say, “Are you the only one with this problem? Why do you complain about everything that happens between you and your husband?” I think the men knowing this tend to maltreat women any way they like. (Naa, 50 years, petty trader, form 4-level education, four children, Krobo)

##### Respecting the Privacy and Sanctity of Marriage

Some participants thought the belief in the privacy and sanctity of marriage influenced women to remain silent. They emphasized that most women are socialized to feel a strong sense of loyalty to their husbands and obligation to the family as a whole, and this translates into keeping abuse private and not involving the police. For these women, the choice to live with sexually abusive husbands was tied to cultural and religious beliefs about the sanctity of marriage as an institution to be protected from public scrutiny. The following comments are illustrative:

It is sometimes difficult to report your husband to the police or to the social welfare unit because he forced to have sex with you. It makes people know what is going on in your marriage, and then they begin to gossip. People even begin to point hands at you when they see you in town, calling you all sorts of names. Sometimes when you think of all these, you just do not feel like going to report your husband to the police or the social welfare unit. (Efua, 37 years, teacher, secondary education, two children, Krobo)

He slapped me when I didn't want to have sex with him. My heart began to beat very fast because I was not expecting this. I was shocked by what he did, and I became depressed. But I could not report him to the police because everyone would get to know the problems in your family. I only reported him to my uncles. They called my husband and warned him never to touch me again, but later, my mother called me and advised me against discussing my marriage issues with people. She said it is not good for a woman to be discussing her marriage issues with people. (Aku, 37 years, petty trader, no education, three children, Krobo)

#### Psychological Barriers and Childhood Abuse and Neglect Issues

##### Fear of Marriage Breakdown

Participants identified the fear of marriage breakdown as a reason for not reporting sexual assaults to law enforcement agencies. Some women said the traditional values of marriage prevented them from disclosing their sexual violence experiences to the police. Despite severe pain following abuse, they wanted to keep their marriages intact and avoid outside intervention:

I never reported him to the police or the women and juvenile unit. I wanted to, especially when he threatened to stab me with a knife, but the people around discouraged me. They said if I go and report him, he will be put behind bars, and he was going to develop hatred for my children and was not going to take care of the children. They said I could go and report him if only I am ready to give up my marriage because he was not going to marry me when he is released from the cells. (Fuseina, 48 years, petty trader form 4-level education, five children, Krobo)

In their attempts to protect their marriages and families from outside intervention, some women gave excuses for their husbands’ violent acts:

He is my husband, and I am living in the same room with him, so I cannot report him just because of little problems. I see it as the works of the devil, so we have to drive the devil out of the house. Even with the big issue we had, people were saying I should go and report to the police, but I did not see it as wise to do that because arresting him means the end of the marriage. He even said he did not know what pushed him to do that, meaning it was a spirit, so he should have solved the problem at that time, and so we should drive the enemy out. So, when I think of all these, I become disturbed and cannot sleep. (Jamilatu, 43 years, petty trader, form 4-level education, three children, Krobo)

##### Concerns About Shame and Stigma

Participants mentioned socio-cultural norms and attitudes as a main reason for not reporting sexual assaults. The majority of the participants explained that traditional norms and values underestimate the severity of sexual assaults and make survivors vulnerable to shame, blame, and stigma. The shame of disclosing sexual assaults often made survivors reluctant to seek help:

I was too shy to tell anyone that my husband forced to have sex with me. So, I kept it to myself and was really suffering within. It is never a good situation to go through. It has many effects on the human body. It could lead to several sicknesses or death of the victim. It got to a point where I thought it would have been better if I were dead. God saw me through this situation. (Ayishetu, 38 years, petty trader, four children, Krobo)

## Discussion

While sexual and domestic violence against women has gained attention in sub-Saharan Africa in recent decades, research on help-seeking remains limited, especially work focusing on barriers to help-seeking among women experiencing sexual violence in their marriage. Yet, the literature suggests these women are not only suffering cumulative male partner sexual violence but may also be facing multiple other challenges, such as financial difficulties, that prevent them from reporting the violence to law enforcement agencies (Adinkrah, 2011; Adu-Gyamfi, 2014; Apatinga & Tenkorang, 2020). By applying the barriers model to explore help-seeking among Ghanaian women who have suffered sexual violence in their marriages, this study adds to the sparse literature on sexual violence within intimate relationships.

Findings show male partner sexual violence was commonplace among the participants and several overlapping barriers undermined their ability to seek help. Barriers in the environment included financial difficulties and misgivings about the justice system. Other barriers were related to family/socialization/role expectations and psychological consequences such as stigma and shame. The findings support the barriers model's contention that female survivors face numerous challenges at multiple scales to reporting violence.

### Barriers in the Environment

#### High Financial Cost of Help-seeking

In agreement with previous research (Barnett et al., 2016; Gharaibeh & Oweis, 2009; Issahaku, 2012), financial difficulties hindered help-seeking among the study's participants. As explained by the barriers model, financial difficulties can trap women in abusive relationships. The majority of our participants counted on low-paying jobs for survival. A lack of financial resources meant they could not raise the money they needed to seek justice, including paying for travel to a police station. Their poverty resulted in financial dependence on abusive husbands and affected their help-seeking behavior. Most were more concerned about meeting their basic needs than seeking help. In such situations, husbands’ financial contributions to the household may be crucial. Women may stay married to continue receiving financial support even when their husbands are abusive. In fact, a significant number of participants in this study talked about financial dependence on their husbands as an impediment to seeking help. This finding highlights the need to economically empower women to enable them to come forward and report sexual and domestic violence.

### Child Support and Well-being

The well-being of children was another barrier to seeking help mentioned by our participants and was closely related to the financial barrier mentioned above. Poverty is widespread in the Lower Manya Krobo municipality and elsewhere in Ghana, making most women dependent on their husbands for financial assistance. If husbands are imprisoned, women may be incapable of providing for their children. The barriers model suggests the lack of shelter, support systems, employment opportunities, and safe housing may undermine women's help-seeking behaviors. Some participants also feared losing custody of their children and did not want them to suffer at the hands of other women or be left without future support (Hassouneh-Phillips, 2001). Overall, the women in this study, regardless of their experiences, were reluctant to press charges against their abusive husbands because of the welfare of their children. Other studies have similarly found children can be a barrier to help seeking (Leonardsson & San Sebastian, 2017; Pinnewala, 2009). However, in Bangladesh, Parvin et al. (2016) had different results.

#### Concerns About the Justice System

Participants expressed misgivings about the legal system, especially the police force. The barriers model theorizes that when abused women lack access to resources and opportunities such as police assistance, it may be difficult to report violence. In Ghana, access to justice is a problem for many, especially the poor and marginalized, because of the unequal distribution of resources (Agbitor, 2012). Corruption and bureaucracy may also stop victims, especially those from poor families, from seeking justice (Sunshine & Tyler, 2003; Tankebe, 2008, 2009). The lack of confidence in the legal system alone may provide a basis for not seeking help. Evidence shows Ghanaians distrust the police force and doubt the objectivity of police procedures (Boateng, 2015; Tankebe, 2009). Some participants declined to report their abusive husbands because they believed their husbands’ financial resources would give them more power to influence the process. Accordingly, some settled violent cases through pastors, chiefs, and elders in the community. However, the failure of women to file complaints to legal authorities may motivate some husbands to continue to perpetrate sexual violence.

### Barriers Due to Family/Socialization/Role Expectations

#### Lack of Social Support

In line with previous research (Benoit et al., 2015; Odette, 2012; Sedziafa et al., 2016), the study's participants mentioned a lack of social support as a barrier to help-seeking. The barriers model considers the lack of social support as central in trapping victimized women in abusive relationships. An essential requirement is the availability of reliable social support and the knowledge that others are willing to provide assistance. However, in this study, the participants’ formal help-seeking intentions were hindered by the absence of social support. Notably, family members and friends discouraged women from seeking formal help, possibly because of the importance attached to marriage in traditional African societies. It could also be possible that family members did not want to bring shame upon themselves and, therefore, chose to counsel women not to formally pursue justice. Family members or friends may not wish to expose family problems to outsiders. The findings suggest societies and cultures where victims lack social support may have higher rates of non-disclosure of sexual violence and abuse. In addition, some men may capitalize on the lack of social support to perpetrate violence when there are intra-household conflicts and disagreements.

#### Respecting the Privacy and Sanctity of Marriage

Consistent with previous studies (Chireshe, 2015; Ellison et al., 2007), respect for the privacy and sanctity of marriage impeded our participants’ help-seeking behavior. The barriers model posits that belief systems function as significant impediments to reporting violence. Participants respected the institution of marriage, and some were advised by relatives and friends to keep family matters secret. This belief discouraged survivors from seeking help. A feature of many African societies is that the welfare of the family unit is valued more than the welfare of the individual; family interests transcend the individual's interests. Accordingly, participants endured pain in silence. However, remaining silent about domestic abuse can promote a culture of violence.

### Psychological Barriers and Childhood Abuse and Neglect Issues

#### Fear of Marriage Dissolution

The fear of marriage breakdown was associated with non-help-seeking among the participants. In Africa, women are socialized to feel responsible for protecting their families’ integrity and solving marital problems. They are expected to play important roles in building and protecting their families because of their traditional role as home keepers or caregivers. This responsibility may make women accept blame and feel guilty for violence in their marriages; this, in turn, may have implications for self-esteem and make it difficult for them to escape abuse. Some participants said they wanted to keep their marriages intact, and this prevented them from seeking help. They attached importance to their marriages and did not want to be divorced or separated from their husbands. This finding corroborates observations made in other studies (Sedziafa et al., 2016; Yigzaw et al., 2010).

#### Stigma and Shame

Finally, the fear and consequences of stigma and shame contributed to participants’ unwillingness to seek help from formal authorities. The barriers model identifies fear and psychological consequences as significant determinants of non-help-seeking behavior. Many survivors worry about the long-term consequences of reporting violence. In Ghana and sub-Saharan Africa, marriage is considered not just private but also sacred; thus, reporting domestic violence to law enforcement agencies may be considered sacrilegious in more religious communities. The stigma of domestic violence can taint victims and perpetrators alike. When sexual violence is exposed publicly, especially involving married couples, it may be ridiculed. Anecdotal evidence indicates that religious homes are considered safe places devoid of domestic violence; reporting sexual violence would affect this perception, contributing to the silence on the issue in a highly religious community like Lower Manya Krobo. The need to protect family integrity is essential, causing survivors to remain silent. Some of our participants were reluctant to seek help because of the desire to protect their image and the image of their families. Other scholars have also found that shame and stigma affect the help-seeking behaviors of women survivors of domestic violence (Chireshe, 2015; Issahaku, 2012; Sedziafa et al., 2016).

## Implications of the Findings

This study, to the best of our knowledge, is the first in Ghana to examine barriers to help-seeking among women who have suffered sexual violence in their marriages. The findings will provide researchers and policymakers in Ghana with insights into why married or cohabiting women do not report sexual abuse to law enforcement agencies. As this study shows, non-disclosure of sexual violence among married or cohabiting women is commonplace in the Ghanaian context, and there are multiple intersecting barriers to help-seeking. This calls for immediate interventions, as the consequences of non-help-seeking can be devastating for survivors and the wider society. The perpetration of sexual violence against married women is a public health issue; it can hinder socio-economic development and prevent the attainment of the Sustainable Development Goal (SDG) of eliminating gender-based violence. Thus, it is important to create community sensitization programs to empower victimized women to seek help.

This study will raise awareness about barriers to help-seeking among women who experience sexual violence in marriage and encourage further research on the subject matter in different socio-cultural contexts. Expanding knowledge and disseminating new information on the topic may lead to better application of existing laws and encourage governments and policymakers to tackle this problem.

## Strengths and Limitations of the Study

As this study is the first of its kind to document the experiences and perspectives of barriers to reporting sexual violence among married or cohabiting women in Ghana using the barriers model, it augments and complements intellectual discussions and the sparse literature on sexual violence within intimate relationships. However, the findings cannot be generalized to all married or cohabiting women who are sexually abused because of the small sample size. Women's narratives on the barriers to reporting sexual violence may vary widely across space and time. Thus, the results do not represent the larger social context. The study sheds light on the barriers to reporting sexual violence among married or cohabiting women, but dedicating large-scale empirical research to this subject across different ethnic and cultural groups would significantly improve our understanding of the phenomenon. Regardless of the aforementioned limitations, the results suggest the need for immediate scholarly and policy attention in Ghana.

## Conclusion

Using qualitative data with the barriers model, this study explored barriers to help-seeking among a sample of Ghanaian women who had experienced male partner sexual violence. Most had not reported sexual assaults to law enforcement agencies, and they cited social, economic, cultural, and legal reasons. Non-help-seeking among sexually abused married or cohabiting women exposes them to intersecting vulnerabilities such as psychosocial health problems and may motivate abusive male partners to continue to perpetrate the violence. Thus, there is a need to implement policies and programs to enable women to seek help. Improving existing laws and their implementation would be instrumental in empowering married or cohabiting women to report sexual violence and abuse. For instance, criminalizing marital rape explicitly in Ghana's Domestic Violence Act (732) may help survivors of sexual violence seek help without fear.

We should highlight, however, that the patriarchal culture in Ghana and women's socialization to believe male partners have unlimited sexual access remain barriers to reporting marital rape, even if the law criminalizes this act (Sedziafa et al., 2019). Under conditions where women subscribe to these patriarchal norms, it is possible they will comply with marital rape instead of resisting. Dealing with this requires changing norms around masculinity; we need an approach that engages male partners in public education and sensitization around sexual abuse and rape within marriage and relationships.

The findings also reveal that multiple intersecting factors at various levels prevent female survivors of abuse from seeking help from law enforcement agencies. This suggests that promoting reporting behavior among married or cohabiting women would require adopting multi-level preventive strategies. Law enforcement agencies, faith-based organizations, non-governmental organizations, the media, and civil society organizations must work collaboratively by sharing information and resources to educate women on the significance of reporting sexual and domestic abuse. Coupled with this, planning community-level programs such as education to engender change in stereotypical gender norms and behaviors, and providing women with economic opportunities to improve their social and economic status, will help empower them to come forward and report sexual and domestic violence.

## Notes

1. Bride price is a payment made by a man or his family to a woman's family during the marriage ceremony. While in some cultures, animals or properties are presented to the woman's family, in others monetary payments are customary. This custom is practiced in many parts of Africa, Asia, the Middle East, and some Pacific Island cultures.

2. Dumase-Krobo is a local area in the Lower Manya Krobo District (study area) in the Eastern Region of Ghana.

3. Akuse is a town in the Lower Manya Krobo District (study area) of the Eastern Region of Ghana. It has a number of public institutions, including the Ghana police service and the Ghana health service.
